# Differences in physical environmental characteristics between adolescents’ actual and shortest cycling routes: a study using a Google Street View-based audit

**DOI:** 10.1186/s12942-018-0136-x

**Published:** 2018-05-29

**Authors:** Hannah Verhoeven, Linde Van Hecke, Delfien Van Dyck, Tim Baert, Nico Van de Weghe, Peter Clarys, Benedicte Deforche, Jelle Van Cauwenberg

**Affiliations:** 1 Department of Public Health, Faculty of Medicine and Health Sciences, Ghent University, Corneel Heymanslaan 10, 9000 Ghent, Belgium; 2 Physical Activity, Nutrition and Health Research Unit, Faculty of Physical Education and Physical Therapy, Vrije Universiteit Brussel, Pleinlaan 2, 1050 Brussels, Belgium; 3 Research Foundation - Flanders (FWO), Brussels, Belgium; 4 Department of Movement and Sport Sciences, Faculty of Medicine and Health Sciences, Ghent University, Watersportlaan 2, 9000 Ghent, Belgium; 5 Department of Geography – CartoGIS, Faculty of Sciences, Ghent University, Krijgslaan 281, 9000 Ghent, Belgium

**Keywords:** Active transport, Cycling, Route choice, Physical environment, Audit, Youth

## Abstract

**Background:**

The objective evaluation of the physical environmental characteristics (e.g. speed limit, cycling infrastructure) along adolescents’ actual cycling routes remains understudied, although it may provide important insights into why adolescents prefer one cycling route over another. The present study aims to gain insight into the physical environmental characteristics determining the route choice of adolescent cyclists by comparing differences in physical environmental characteristics between their actual cycling routes and the shortest possible cycling routes.

**Methods:**

Adolescents (n = 204; 46.5% boys; 14.4 ± 1.2 years) recruited at secondary schools in and around Ghent (city in Flanders, northern part of Belgium) were instructed to wear a Global Positioning System device in order to identify cycling trips. For all identified cycling trips, the shortest possible route that could have been taken was calculated. Actual cycling routes that were not the shortest possible cycling routes were divided into street segments. Segments were audited with a Google Street View-based tool to assess physical environmental characteristics along actual and shortest cycling routes.

**Results:**

Out of 160 actual cycling trips, 73.1% did not differ from the shortest possible cycling route. For actual cycling routes that were not the shortest cycling route, a speed limit of 30 km/h, roads having few buildings with windows on the street side and roads without cycle lane were more frequently present compared to the shortest possible cycling routes. A mixed land use, roads with commercial destinations, arterial roads, cycle lanes separated from traffic by white lines, small cycle lanes and cycle lanes covered by lighting were less frequently present along actual cycling routes compared to the shortest possible cycling routes.

**Conclusions:**

Results showed that distance mainly determines the route along which adolescents cycle. In addition, adolescents cycled more along residential streets (even if no cycle lane was present) and less along busy, arterial roads. Local authorities should provide shortcuts free from motorised traffic to meet adolescents’ preference to cycle along the shortest route and to avoid cycling along arterial roads.

**Electronic supplementary material:**

The online version of this article (10.1186/s12942-018-0136-x) contains supplementary material, which is available to authorized users.

## Background

Air pollution, which is partially caused by vehicle emissions, is consistently related to acute respiratory infections among young children, cardiopulmonary disease and lung cancer [1]. By replacing private car use (passive transport) by active modes of transport such as cycling, carbon dioxide emissions can be reduced substantially [2]. Although the risk of a higher intake of carbon dioxide can be considered as a negative aspect of active transport [3], a growing body of evidence emphasizes the potential benefits of cycling for transport for public health [2, 4]. Since adolescence is characterised by a steep decrease in physical activity levels [5], increasing cycling for transport is also a promising strategy to meet the recommended 60 min of daily physical activity among adolescents [4, 6]. Cycling for transport has been associated with higher levels of cardiorespiratory fitness [7] and lower levels of overweight [8] among adolescents and it can easily be incorporated into their daily lives once the skills for cycling have been acquired [9].

The role of the physical environment for health behaviours such as cycling for transport has been acknowledged by socio-ecological models and previous research [10–12]. However, the majority of previous studies investigating physical environmental correlates of cycling for transport focused on the neighbourhood environment close to home, although cycling for transport does not necessarily take place in the immediate neighbourhood environment. Nevertheless, the evaluation of physical environmental characteristics along adolescents’ actual cycling routes remains understudied, although it is important to find out why individuals chose a specific cycling route. In addition, although previous studies emphasized the importance of distance for adolescents’ cycling for transport [12–14], it is likely that adolescents do not always take the shortest cycling route. By comparing adolescents’ actual cycling routes with the shortest possible cycling routes, important information regarding which physical environmental characteristics determine the route choice of adolescent cyclists may be obtained. Among adults, two recent studies compared physical environmental characteristics of actual and shortest cycling routes [15, 16]. Winters et al. [16] found that actual cycling routes of Canadian adults had significantly more traffic calming facilities (e.g. traffic circles or median barriers to slow or block motorized traffic) and participants cycled less along arterial (busy) roads and more along local roads, off-street paths and roads with cycling facilities. Krenn et al. [15] also found that Austrian cyclists avoid busy roads and prefer roads with cycle lanes. Actual cycling routes included more green and aquatic areas and had fewer traffic lights, fewer crossings and less hilly roads compared to the shortest routes. Compared to the shortest routes, land use mix (i.e. the extent to which several types of land use, such as residential and industrial areas, shops, services, are included in an area) was significantly higher along actual cycling routes. A study among children in the Netherlands (8–12 years) found that there were significantly fewer trees, zebra crossings and sidewalks along actual cycling routes compared to the shortest routes [17]. In addition, actual cycling routes had significantly more traffic lights, junctions and a higher chance of being on residential streets compared to the shortest routes. Safety showed thus to be an important factor among children in this study. According to Dessing et al. [17], most of the zebra crossings in the Netherlands are located on or near busy streets, that were avoided by the children. Furthermore, when main roads have to be crossed children preferred signalized intersections. Because of some inconsistent results across these previous studies, similar studies among adolescents may provide additional insights into which physical environmental factors are related to an individuals’ route choice.

Methodologies to assess the physical environment include both subjective and objective measurements. Subjective measurements, such as self-reported questionnaires, encounter limitations such as recall bias [18] and may not accurately assess the effect of the actual physical environmental factors on cycling for transport [11]. Therefore, observational field audits are frequently applied as an objective tool for measuring the physical environment related to physical activity [19–21]. Vanwolleghem et al. [22] developed EGA-Cycling (Environmental Google Street View Based Audit-Cycling) to virtually assess physical micro- and macro-environmental characteristics along cycling routes using Google Street View. EGA-Cycling was based on existing audit instruments (e.g. Pikora-SPACES instruments [20], Audit Tool Checklist version [21], Irvine-Minnesota Inventory [23]), but was adapted to the Flemish street infrastructure. In the last decade, using virtual technologies, such as Google Street View, to assess the physical environment is gaining attention [24–29]. Auditors are able to virtually walk through a street which is time- and cost-saving [24, 28] and they are not exposed to unsafe (traffic) situations compared to field audits. Previous studies showed good agreement between virtual and field audit tools [24, 26, 29]. However, virtual audit tools showed to be less accurate when measuring micro-environmental characteristics (e.g. litter, sidewalk condition) [24, 26, 28]. Nevertheless, Ben-Joseph et al. [28] concluded that Google Street View was more accurate in measuring small features compared to Google Maps and MS Visual Oblique.

The aim of the present study is to gain insight into the physical environmental characteristics determining the route choice of adolescent cyclists by comparing differences in physical environmental characteristics between their actual cycling routes and the shortest possible cycling routes using a Google Street View-based audit (EGA-Cycling).

## Methods

### Participants

A convenience sample of 12 secondary schools in and around Ghent was contacted to participate in the study. Ghent is a city in Flanders, northern part of Belgium, that has 253,266 inhabitants and comprises an area of 156.2 km^2^ (population density: 1622 h/km^2^) [30, 31]. In the six schools that agreed to participate, school principals or staff members randomly selected at least two classes from the first to fourth grade (12–16 years). A total of 18 classes was selected and 283 adolescents were invited to participate in the study. Only participants who were present at school when measurement materials were handed out, could be included in the study. Passive informed consent was obtained from adolescents’ parents. If parents did not agree to let their child participate in the study, they had to sign a form. Furthermore, researchers also obtained active informed consent from adolescents. This procedure resulted in a group of 238 adolescents (response rate = 84.1%) participating in the study.

### Study protocol

**Fig. 1:**
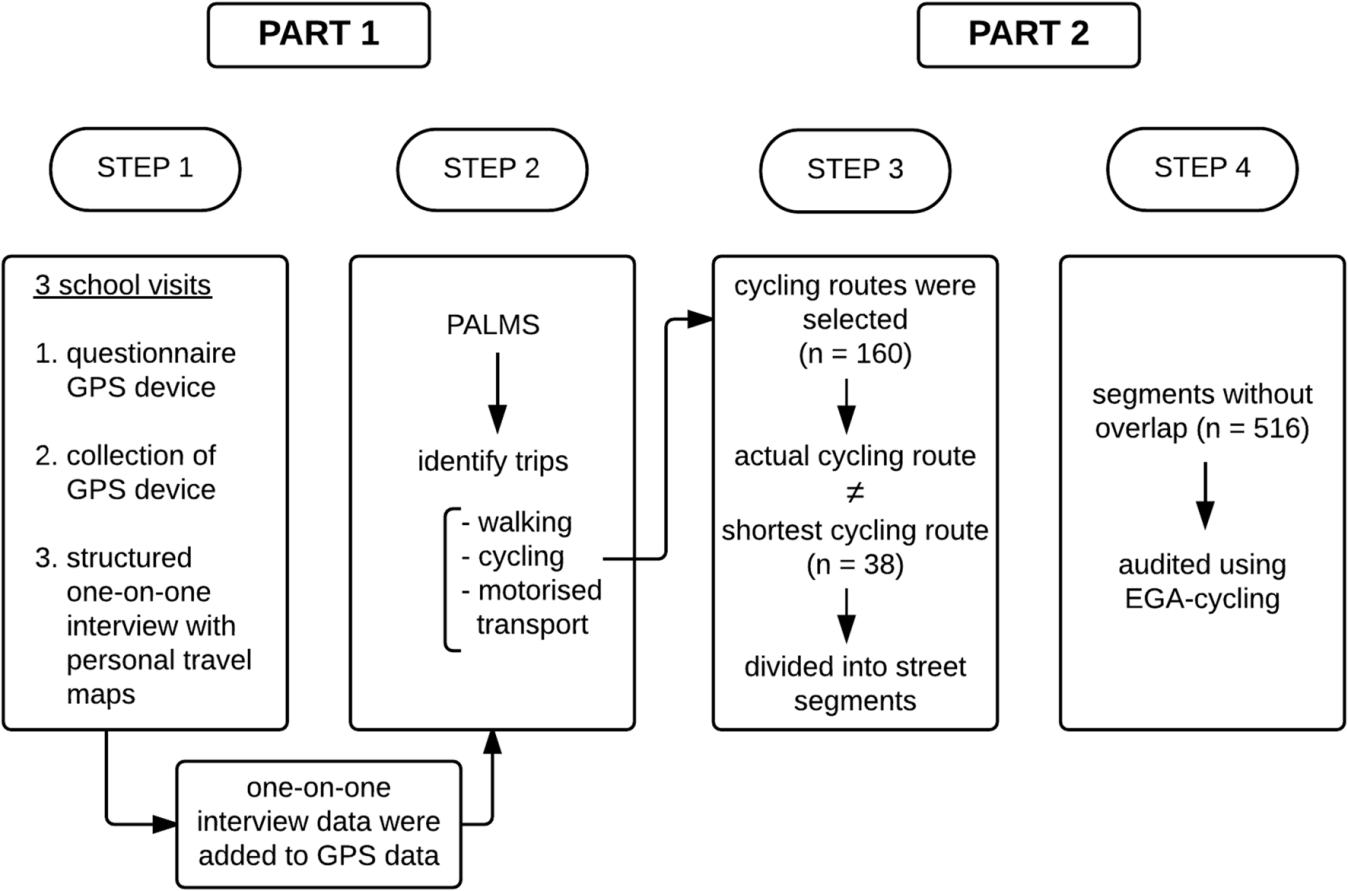
Flow chart

The study protocol consisted of two parts (see Fig. 1 for a flow chart). In the first part of the study, each participating school was visited three times by the research team between September and December 2015. During a first visit, the purpose of the study was explained to the adolescents and informed consent was obtained. Each participant received a unique ID number in order to be able to link data of all measurements. Participants completed a questionnaire assessing socio-demographics. Furthermore, participants received a Global Positioning System (GPS) device and a charger for the device together with verbal and written instructions on how and when to wear the device. All participants were instructed to wear the GPS device, which was attached to their waist with an elastic belt, during waking hours until the research team returned to the school to collect the devices (4–5 days later). During activities that could damage the GPS device or during which it could be uncomfortable to wear it (e.g. showering, swimming or rugby), the adolescents were asked to temporarily remove the GPS device. They were also instructed not to turn off the GPS device during data collection. Participants were asked for their mobile phone number. Two text messages per day (in the morning and evening) were sent to the participants willing to give their number in order to remind them to wear the GPS devices and to charge it. During a second visit, researchers returned to the schools to collect the devices. Afterwards, the GPS data were downloaded and a web application was created in order to visualize the data on a personal travel map. During the last visit, which took place within the first week after collection of the GPS devices, researchers conducted a structured one-on-one interview (Additional file 1) during which a researcher chronologically discussed the personal travel maps. Per trip travelled, participants were asked about their transport mode and why they took a particular route. Participants who completed all measurements and returned the GPS device received an incentive (i.e. movie ticket).

In the second part of the study, adolescents’ cycling routes were selected, and for each actual cycling route the shortest cycling route was calculated using Google Maps. For each cycling route which was not the shortest cycling route, an adapted version of EGA-Cycling was used to obtain information about physical environmental characteristics along adolescents’ actual cycling routes and along the corresponding shortest routes using Google Street View.

The study protocol was approved by the Ethics Committee of the University Hospital of Ghent University (EC 2015/0317).

### Measurements and data processing

#### Questionnaire

Participants completed a paper-and-pencil questionnaire assessing following socio-demographics: home address, gender, date of birth, grade (first to fourth year), educational type (general, technical or vocational) and highest education of parents (primary education, secondary education, tertiary education-non university, tertiary education-university, I don’t know). Education of parents was used to as a proxy for socio-economic status (SES). Adolescents were identified as being ‘of a higher SES family’ when at least one parent completed tertiary education [32].

#### GPS device

The geographical position of participants was recorded by the QStarz BT-Q1000X GPS device. In addition, the GPS device recorded participants’ speed which was used to define their transport mode [33]. The GPS devices were set to collect data every 30 s using Q-travel software. Furthermore, the devices were set to stop logging when the memory was full (this did not occur during data collection). Q-travel software was used to download the collected GPS data.

#### Structured one-on-one interview with personal travel maps

**Fig. 2:**
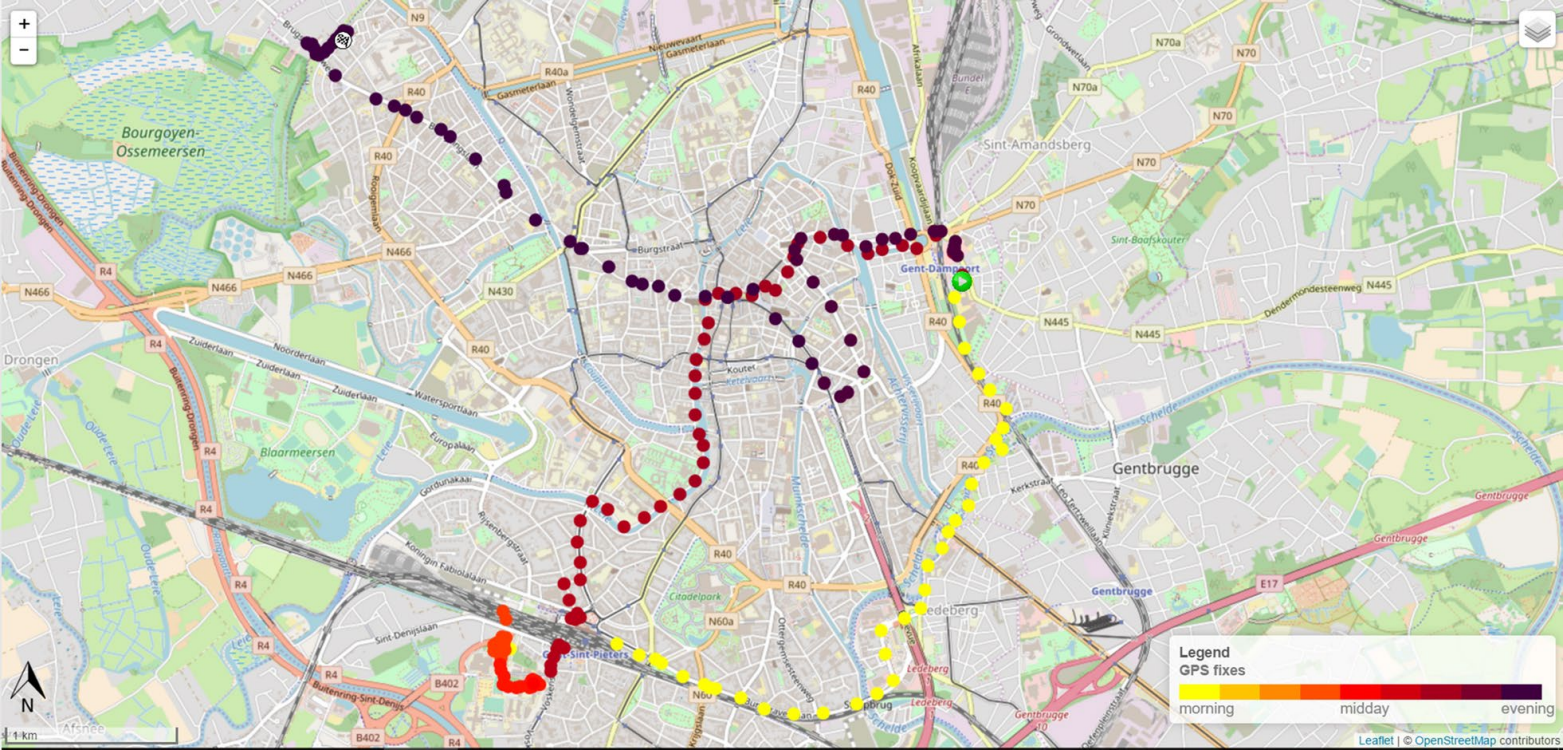
Example of a personal travel map. Every 30 s a dot was placed on the map (temporal resolution: 30 s). The green arrow represents the first data point of the day registered by the GPS and the ‘finish flag’ represents the last registered data point of the day by the GPS

GPS data were stored in a PostgreSQL database with PostGIS in order to generate a personal travel map per day in the web application. This web application showed the geographical position of participants for every 30-second-interval. Figure 2 shows an example of a personal travel map. These personal travel maps were used as a guide to conduct a structured one-on-one interview discussing routes on two selected days. The first week- and weekend day with complete data (excluding the day the devices were handed out) were selected for the interview. When no weekdays with complete data were available, two weekend days were selected and vice versa. When only 1 day with complete data was available, the structured interview was completed for 1 day. During these interviews, a researcher chronologically identified, together with the participants, the trips they made during a day. For each identified trip, the participant was asked which transport mode was used. For active trips (walking or cycling/skateboard/…) the participant was also asked why he/she chose that particular route to reach his/her destination.

#### GPS data processing

Data processing was executed using the Personal Activity and Location Measurement System (PALMS©) [34, 35]. PALMS filtered invalid GPS data when extreme speed (> 150 km/h) or extreme changes in distance (> 1000 m) or elevation (> 100 m) between two consecutive data points were identified. The programming software Python was used to combine the PALMS dataset with information on school schedules of each participating class, school addresses and home addresses of participants. PALMS categorised data into location (home, school, leisure) or transport. Data were categorised in the domain ‘transport’ when a trip was detected. A trip was defined as a period of at least 3 min of movement with the same transport mode, allowing for stationary periods of maximum 3 min. PALMS classified all trips into walking, cycling or motorised transport based on speed. A trip was classified as walking when speed was between 1 and 9 km/h, cycling between 10 and 24 km/h and motorised transport starting from 25 km/h [33, 36].

Subsequently, all data from the structured one-on-one interviews were inserted into the database. For trips or locations that were misclassified by PALMS [e.g. when a car trip was classified as a bicycle trip due to traffic congestion (speed < 25 km/h)], corrections were made based on the data of the structured interviews. The number of corrections due to misclassification by PALMS was rather limited.

#### EGA-cycling

EGA-Cycling (Additional file 2) consists of five subscales and includes 37 items: (1) land use (8 items; e.g. commercial destinations, heavy industry and public destinations), (2) general characteristics of the street segment (12 items; e.g. road type and speed limit), (3) cycling facilities (7 items; e.g. type and width of cycle lane), (4) pedestrian facilities (3 items; e.g. presence and maintenance of the sidewalk) and (5) aesthetics (7 items; e.g. trees and front yards). EGA-Cycling shows acceptable reliability and validity [22]. However, since measures about (safety at) intersections are very limited from this tool, three additional items regarding this topic were added (i.e. amount of side streets, amount of intersections and visibility at the corners). The item regarding visibility at the corners is part of the Microscale Audit of Pedestrian Streetscape (MAPS) Global tool [37]. Furthermore, another item was included that assessed whether or not the street segment concerned a walking/cycling road (i.e. a separate road only accessible for non-motorised traffic). Data on differences in pedestrian facilities between actual cycling routes and the shortest possible cycling routes are not shown since they are not relevant for cycling.

#### Auditing of actual and shortest cycling routes

**Fig. 3:**
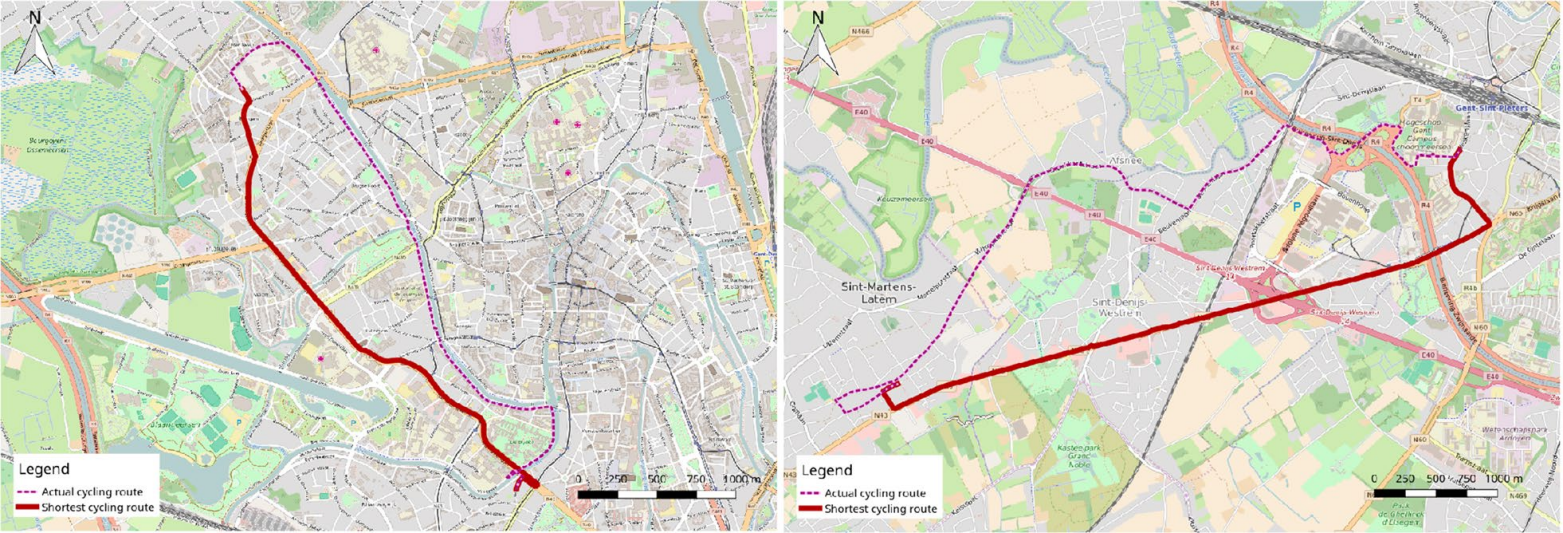
Examples of actual versus shortest cycling routes

For all cycling trips that could be identified in the previous steps, the shortest cycling route was calculated using Google Maps. Only actual cycling routes that were not the shortest possible cycling routes were selected to be used in subsequent analyses. All routes for which the actual cycling route was not the shortest possible cycling route were included, even if only one segment differed between the actual and the shortest cycling route. Differences in distance between actual cycling routes and the shortest possible cycling routes were calculated absolutely in meters as well as relatively in percentage of the shortest cycling route (reported as ‘detour’). Google My Maps (a Google Maps application) was used to visualize actual cycling routes and the corresponding shortest cycling routes. Each cycling route was manually divided into several street segments (average distance: 342 ± 468 m), a new street segment started when participants turned into another street or when the street name changed. For each street segment, EGA-Cycling was filled out by one out of three trained observers (the first author and two independent observers). Google Street View was used to perform the audits, which took approximately 2 weeks per observer (6 weeks in total). Google Street View images ranged from March 2009 till April 2015. The majority (53.0%) of images were taken between August 2014 and October 2014. Prior to auditing the pre-defined routes, two independent observers were trained by the first author. The training included specific instructions; all items of the EGA-Cycling tool were explained and illustrated with photographs if necessary. Subsequently, the observers audited three random street segments which enabled them to raise questions. Thereafter, five test routes (i.e. no routes that were part of the study) were rated by the first author and the two independent observers. Prior to auditing the pre-defined routes, 95% agreement with the first author’s scores was required. For the actual audits, only street segments for which there was no overlap between the actual cycling route and the shortest cycling route were audited (516 segments). Distances of segments were measured in Google My Maps. Figure 3 shows examples of actual versus shortest cycling routes.

### Data analyses

Data were analysed using IBM SPSS Statistics 24. A paired samples t-test was used to calculate the difference in distance between actual and shortest cycling routes. Because EGA-Cycling was developed to assess physical environmental characteristics along entire cycling routes instead of individual segments [22], a total score per cycling route was calculated for each item. Per item, the score for a particular segment was multiplied by the distance of that segment. These weighted item scores of several segments of a route were summed to obtain one total score per route for that item. Subsequently, item scores were expressed in m/km in order to be able to compare the actual cycling route with the shortest cycling route (for which the route length differed). Univariate multilevel logistic regression analyses were used to investigate differences in physical environmental characteristics between actual and shortest cycling routes (three levels: participant, route and street segment). Statistical significance was set at p < 0.05.

## Results

### Sample characteristics

From the 238 adolescents participating, adolescents older than 17 years (n = 4) and participants who did not wear/charge the material properly (n = 13) were removed from the dataset as were participants who were absent when the structured interviews were completed (n = 17). A final sample of 204 adolescents (85.7%) was used for data analyses (46.5% boys, 14.4 ± 1.2 years). Table 1 presents descriptive characteristics of the sample. Within this sample, a total of 1126 trips was identified. Passive transport (car, as a passenger) was used most frequently (34.6% of trips), followed by public transport (33.9% of trips). Active transport such as walking and cycling was used for 17.2 and 14.2% of trips, respectively. The purpose of a trip and the transport mode used showed to be related to each other (Chi^2^ = 257.1; p < 0.001). For trips to and/or from school, the majority (57.2%) was done by public transport, 18.4% was done by bicycle, 12.6% by foot and 11.8% by passive transport. For leisure-related trips, nearly half (49.6%) was done by passive transport, 20.1% was done by foot, 17.8% by public transport and 12.5% by bicycle. The median distance for car trips was 6312 m and for public transport a median distance of 4934 m was found. Walking trips had a median distance of 710 m, whereas for cycling trips a median distance of 2633 m was found.

**Table 1:** Descriptive characteristics of the sample (n = 204)

Socio-demographic characteristics	
Gender (% boys)	46.5
Age (years; mean ± SD)	14.4 ± 1.2
Socio-economic status (SES) parents (%)	
Lower SES (% no parent completed tertiary education)	28.4
Higher SES (% at least one parent completed tertiary education)	71.6
Grade (%)	
1st year of secondary school	8.3
2nd year of secondary school	7.4
3rd year of secondary school	46.1
4th year of secondary school	38.2
Educational type (%)	
General education	65.2
Technical education	10.3
Vocational education	24.5

**Fig. 4:**
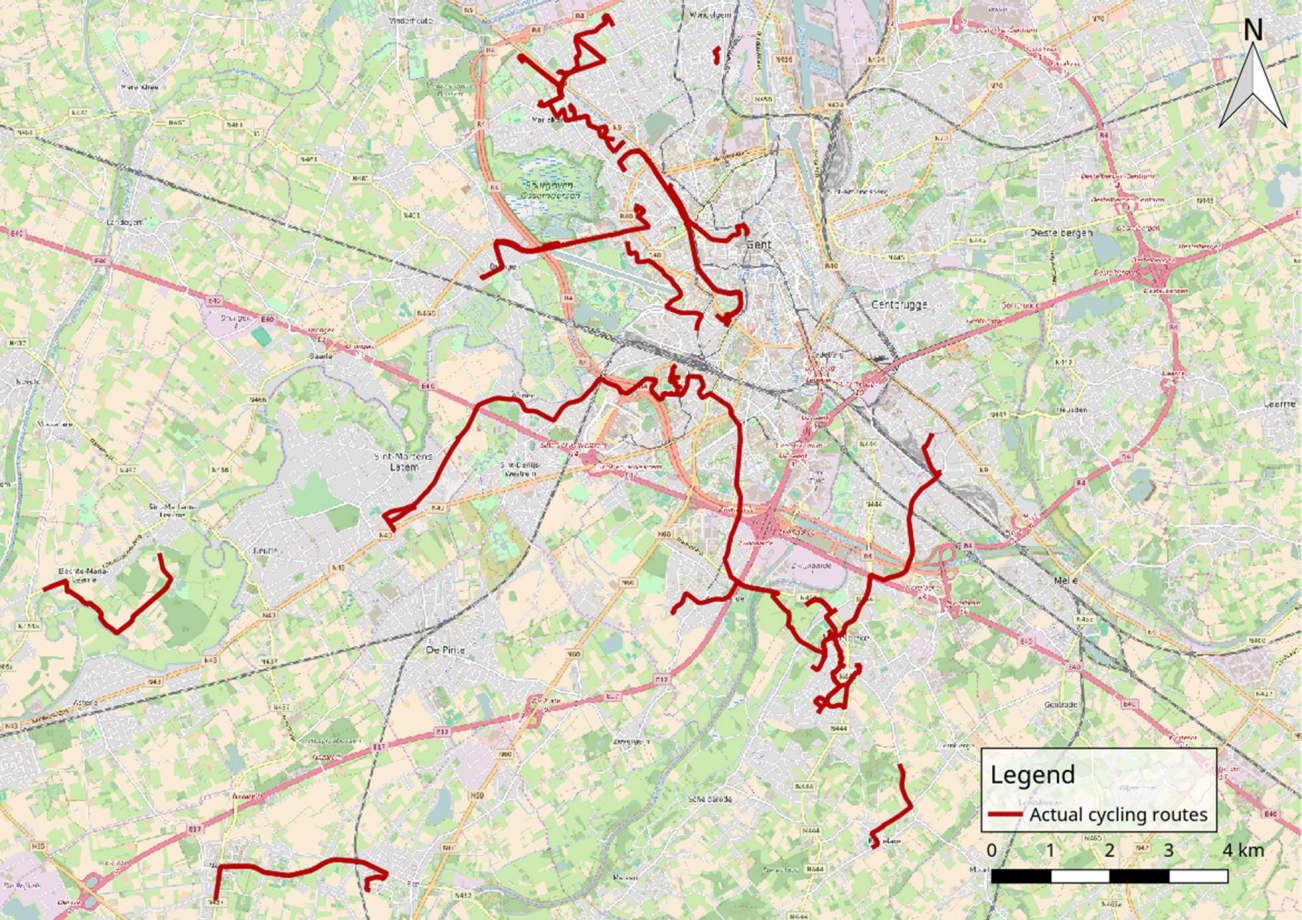
Overview of the 38 actual cycling routes that differed from the shortest possible cycling route

Out of 160 actual cycling trips, 73.1% did not differ from the shortest possible cycling routes. Thirty-eight unique cycling routes for which the actual route differed from the shortest possible cycling route could be identified (see Fig. 4). The 38 routes were spread over 22 adolescents, with a range of 1 to maximum 4 routes per person. A significant difference in distance between actual and shortest possible cycling routes was found (t = 8.606; p < 0.001). Actual cycling routes had a mean distance of 4505 ± 2201 m (med = 4100 m; min = 1000 m; max = 8800 m), whereas for the shortest possible cycling routes a mean distance of 3989 ± 2048 m (med = 3600 m; min = 700 m; max = 8100 m) was found. The mean difference between actual cycling routes and the shortest possible cycling routes (detour) was 516 ± 369 m (med = 400 m; min = 100 m; max = 1600 m). The average detour was 15.6% (med = 12.1%; min = 2.0%; max = 45.7%) in comparison to the shortest possible cycling route.

Table 2 presents results on differences in physical environmental characteristics concerning land use between actual cycling routes and the shortest possible cycling routes. An increase in 100 m/km of mixed land use along the actual cycling route, resulted in 16% lower odds that the actual cycling route was chosen over the shortest cycling route. In addition, an increase in 100 m/km where commercial destinations are present along the actual cycling route, resulted in 17% lower odds that the actual cycling route was chosen over the shortest cycling route.

**Table 2:** Presence of items on land use along actual cycling routes compared to shortest cycling routes

Item	Actual cycling route (m/km; M ± SD)	Shortest cycling route (m/km; M ± SD)	OR (95% CI)
Mixed land use	256 ± 226	386 ± 317	0.84 (0.71; 1.00)*
Types of buildings			
Single buildings	155 ± 247	153 ± 256	1.00 (0.83; 1.21)
Closed/semi-detached buildings	225 ± 190	139 ± 182	1.30 (0.99; 1.70)^t^
Apartment buildings	111 ± 200	161 ± 244	0.90 (0.73; (1.12)
Commercial destinations	233 ± 229	367 ± 304	0.83 (0.69; 0.99)*
Heavy industry	9 ± 39	17 ± 63	0.74 (0.29; 1.91)
Public destinations	248 ± 196	355 ± 300	0.84 (0.70; 1.02)^t^
Recreational destinations	85 ± 105	121 ± 212	0.87 (0.65; 1.17)
Natural features	315 ± 314	257 ± 286	1.07 (0.91; 1.25)
Openness view			
Open view	65 ± 174	24 ± 81	1.29 (0.85; 1.96)
Not open/closed view	354 ± 229	370 ± 271	0.98 (0.81; 1.17)
Closed view	166 ± 168	146 ± 185	1.07 (0.82; 1.39)

Reference = shortest cycling route. For ease of interpretation of OR, distances were converted to hectometres (100 m/km)

*OR* odds ratio, *CI* confidence interval

*p ≤ 0.05; **p ≤ 0.01; ^t^p ≤ 0.1

Table 3 presents results on differences in general characteristics between actual cycling routes and the shortest possible cycling routes. An increase in 100 m/km of a road type which consists of two roads divided in two lanes each direction (i.e. arterial road) along the actual cycling route, resulted in 47% lower odds that the actual cycling route was chosen over the shortest cycling route. An increase in 100 m/km with a speed limit of 30 km/h along the actual cycling route, resulted in 50% higher odds that the actual cycling route was chosen over the shortest cycling route. Furthermore, an increase in 100 m/km for roads where few buildings with windows on the street are present along the actual cycling route, resulted in 192% higher odds that the actual cycling route was chosen over the shortest cycling route. This last item refers to crime safety/social control (i.e. if few buildings with windows on the street (or few buildings in general) are present, few people have a clear view on the street and there is thus less social control) [22].

**Table 3:** Presence of items on general characteristics along actual cycling routes compared to shortest cycling routes

Item	Actual cycling route (m/km; M ± SD)	Shortest cycling route (m/km; M ± SD)	OR (95% CI)
Road type			
Walking/cycling road	89 ± 99	62 ± 111	1.29 (0.82; 2.03)
One road for one-direction traffic	120 ± 168	56 ± 107	1.43 (0.97; 2.11)^t^
One road not divided into lanes	259 ± 232	160 ± 195	1.25 (0.99; 1.57)^t^
One road divided in one lane each direction	82 ± 125	123 ± 199	0.86 (0.64; 1.15)
Two roads divided in one lane each direction	21 ± 58	7 ± 24	2.44 (0.63; 9.41)
Two roads divided in two lanes each direction	14 ± 46	130 ± 245	0.53 (0.28; 0.99)*
Speed limit			
30 km/h	145 ± 185	58 ± 131	1.50 (1.02; 2.21)*
50 km/h	309 ± 261	316 ± 255	0.99 (0.83; 1.18)
70 km/h or more	43 ± 87	106 ± 225	0.78 (0.57; 1.08)
Traffic calming measures	248 ± 222	344 ± 304	0.87 (0.73; 1.04)
Amount of side streets	13 ± 11	11 ± 10	1.02 (0.98; 1.07)
Amount of intersections	2 ± 2	3 ± 3	0.90 (0.75; 1.09)
Crossing aids	372 ± 247	414 ± 310	0.95 (0.80; 1.12)
Poor visibility when crossing a street	23 ± 54	4 ± 16	4.85 (0.80; 29.52)^t^
Well-maintained street segment	548 ± 267	531 ± 282	1.02 (0.86; 1.21)
Streetlights	522 ± 265	536 ± 290	0.98 (0.83; 1.16)
Parking facilities			
On street parking facilities	180 ± 173	122 ± 156	1.25 (0.93; 1.68)
Parking facilities next to the street	210 ± 206	321 ± 297	0.84 (0.69; 1.02)^t^
Parking facilities on adjacent parking	18 ± 62	3 ± 15	2.98 (0.44; 20.35)
No parking facilities	88 ± 193	33 ± 55	1.45 (0.86; 2.44)
Slope			
Flat	546 ± 266	499 ± 297	1.06 (0.90; 1.25)
Gentle to moderate slope	39 ± 62	41 ± 105	0.97 (0.56; 1.66)
Swerving alternatives	407 ± 254	353 ± 266	1.08 (0.91; 1.30)
Buildings			
No buildings with windows on street side	47 ± 182	30 ± 82	1.10 (0.78; 1.55)
Few buildings with windows on street side	58 ± 78	22 ± 45	2.92 (1.12; 7.63)*
Many buildings with windows on street side	391 ± 248	427 ± 293	0.95 (0.80; 1.13)
Driveways			
No driveways	54 ± 136	65 ± 105	0.93 (0.63; 1.37)
Approx. 25% of buildings have one driveway	181 ± 207	167 ± 238	1.03 (0.84; 1.27)
Approx. 50% of buildings have one driveway	17 ± 29	35 ± 88	0.61 (0.25; 1.51)
Most buildings have one driveway	244 ± 247	213 ± 259	1.05 (0.88; 1.26)
Garages			
No garages	228 ± 240	187 ± 175	1.10 (0.88; 1.38)
Approx. 25% of buildings have one garage	252 ± 228	270 ± 273	0.97 (0.81; 1.17)
Approx. 50% of buildings or more have one garage	17 ± 32	22 ± 47	0.72 (0.22; 2.32)

Reference = shortest cycling route. Results regarding ‘one road divided in two lanes each direction’ (road type) are not shown since this road type did not appear along the routes. For ease of interpretation of OR, distances were converted to hectometres (100 m/km)

*OR* odds ratio, *CI* confidence interval

*p ≤ 0.05; **p ≤ 0.01; ^t^p ≤ 0.1

Table 4 presents results on differences in cycling facilities between actual cycling routes and the shortest possible cycling routes. For an increase in 100 m/km of a cycle lane which is part of the road (cycle lane separated from traffic by white lines) along the actual cycling route, 36% lower odds that the actual cycling route was chosen over the shortest cycling route was found. For an increase in 100 m/km road with no cycle lane along the actual cycling route, 25% higher odds that the actual cycling route was chosen over the shortest cycling route was found. In addition, an increase in 100 m/km of a small cycle lane along the actual cycling route, resulted in 32% lower odds that the actual cycling route was chosen over the shortest cycling route. Finally, for an increase in 100 m/km of a cycle lane that is covered by lighting along the actual cycling route, 25% lower odds that the actual cycling route was chosen over the shortest cycling route was found.

**Table 4:** Presence of items on cycling facilities along actual cycling routes compared to shortest cycling routes

Item	Actual cycling route (m/km; M ± SD)	Shortest cycling route (m/km; M ± SD)	OR (95% CI)
Type of cycle lane			
Cycle lane separated from the road	74 ± 133	45 ± 75	1.30 (0.82; 2.08)
Adjoining cycle lane (slightly increased)	76 ± 105	89 ± 141	0.91 (0.63; 1.33)
Cycle lane is part of the road (white lines)	52 ± 84	180 ± 252	0.64 (0.44; 0.92)*
Non-compulsory cycle lane or of a different colour	24 ± 101	7 ± 29	1.45 (0.65; 3.25)
No cycle lane	271 ± 243	159 ± 209	1.25 (1.01; 1.56)*
Width cycle lane			
Small	72 ± 106	166 ± 206	0.68 (0.48; 0.96)*
Wide	242 ± 260	237 ± 276	1.01 (0.85; 1.20)
Two-way cycle lane	224 ± 254	121 ± 203	1.23 (0.98; 1.54)^t^
Well-maintained cycle lane	294 ± 240	376 ± 286	0.89 (0.74; 1.06)
Lighting covering cycle lane	174 ± 182	332 ± 284	0.75 (0.61; 0.94)**
Surface cycle lane			
Bitumen	273 ± 177	260 ± 237	1.03 (0.83; 1.29)
Continuous concrete	8 ± 37	5 ± 17	1.50 (0.27; 8.41)
Paving bricks	181 ± 199	126 ± 131	1.22 (0.91; 1.63)
Concrete slabs	80 ± 112	109 ± 181	0.73 (0.64; 1.20)
Cobblestones	12 ± 30	16 ± 44	0.71 (0.20; 2.48)
Gravel	32 ± 77	23 ± 92	1.13 (0.65; 1.98)
Condition cycle lane			
Poor	28 ± 58	18 ± 78	1.25 (0.61; 2.58)
Moderate	223 ± 182	257 ± 258	0.93 (0.76; 1.15)
Good	335 ± 226	264 ± 229	1.15 (0.93; 1.41)

Reference = shortest cycling route. For ease of interpretation of OR, distances were converted to hectometres (100 m/km)

*OR* odds ratio, *CI* confidence interval

*p ≤ 0.05; **p ≤ 0.01; ^t^p ≤ 0.1

Table 5 presents results on differences in aesthetics between actual cycling routes and the shortest possible cycling routes. For none of the included variables, a significant result was found.

**Table 5:** Presence of items on aesthetics along actual cycling routes compared to shortest cycling routes

Item	Actual cycling route (m/km; M ± SD)	Shortest cycling route (m/km; M ± SD)	OR (95% CI)
Trees	459 ± 241	428 ± 294	1.04 (0.88; 1.24)
Attractive buildings	60 ± 111	70 ± 134	0.94 (0.65; 1.37)
Well-maintained buildings	501 ± 240	500 ± 287	1.00 (0.84; 1.19)
Front yards	297 ± 257	328 ± 294	0.96 (0.81; 1.13)
Well-maintained front yards	315 ± 247	398 ± 274	0.88 (0.73; 1.07)
Attractive natural features	250 ± 310	163 ± 243	1.12 (0.95; 1.33)
Graffiti and litter	120 ± 201	93 ± 205	1.07 (0.85; 1.35)

Reference = shortest cycling route. For ease of interpretation of OR, distances were converted to hectometres (100 m/km)

*OR* odds ratio, *CI* confidence interval

*p ≤ 0.05; **p ≤ 0.01; ^t^p ≤ 0.1

Subjective results of the structured one-on-one interviews showed that, for the 38 actual cycling routes that differed from the shortest possible cycling route, adolescents still indicated for 35.1% (n = 13) of the trips that they chose that route because it was the shortest/fastest route. For 16.2% (n = 6) of these cycling trips, participants indicated they chose that particular route to cycle together with friends/siblings/…. For another 16.2% (n = 6) of the trips, they chose that particular route because of lower traffic density. Furthermore, for 13.5% (n = 5) of the trips participants indicated that route choice was determined by their parents and for 5.4% (n = 2) of the trips they indicated to choose that particular route because of the presence of few/safe crossings. For another 5.4% (n = 2) of the trips, participants indicated they chose that particular route because of a commercial destination they wanted to visit.

## Discussion

The present study aimed to investigate differences in physical environmental characteristics between adolescents’ actual cycling routes and the shortest possible cycling routes using a Google Street View-based audit. A mixed land use, roads with commercial destinations, arterial roads, cycle lanes separated from traffic by white lines, small cycle lanes and cycle lanes covered by lighting were less frequently present along adolescents’ actual cycling routes in comparison to the shortest possible cycling routes. Besides, a speed limit of 30 km/h, roads having few buildings with windows on street side and roads without cycle lane were more frequently present along actual cycling routes compared to the shortest possible cycling routes.

In line with previous studies [12–14], the present study showed that a short cycling distance is one of the most important factors determining the route choice of adolescent cyclists, as for 73.1% of the cycling trips participants took the shortest possible route. In addition, for 35.1% of the cycling trips which were not the shortest possible, adolescents still indicated that they chose that route because they perceived it as the shortest/fastest route. Thus, even if a route is not actually the shortest, adolescents may choose this route because they perceive it as the shortest route. For all cycling trips that were not the shortest possible, a mean difference of 516 m (15.6%) between the actual and the shortest cycling route was found. When only looking at those routes which were not the shortest but adolescents perceived as the shortest/fastest route, a mean difference of 431 m between the actual and the shortest cycling route was found, and thus, the detour showed to be smaller. It is possible that adolescents do not notice the difference in cycling time between their actual cycling route they perceive as the shortest and the shortest possible cycling route.

Although some findings of the present study seem to be in contradiction with results of previous cross-sectional studies [12], the findings generally have a clear explanation. The present study showed that adolescents avoid routes with a mixed land use where commercial destinations are present. In contradiction, a US study showed that children and adolescents (5–18 years) were more likely to walk or cycle to school if their parents reported having stores in the neighbourhood environment [38]. However, in accordance with the present study, shops and services were also less present along the actual cycling routes of adults in Austria [15]. Dessing et al. [17] found that children in The Netherlands mainly cycled to school along residential areas to avoid busy streets. These findings are similar to results in our study since residential areas are, in general, characterised by a lower land use mix and less commercial destinations. In the study by Dessing et al. [17], it was suggested that residential streets may be perceived as safe, quiet streets to cycle for transport, even if separate cycle lanes are absent. This could be confirmed by the results of the present study which showed that adolescents mainly cycled along local roads, such as roads for one-direction traffic and roads which were not divided into lanes (trends towards significance), where speed limits of 30 km/h apply. Furthermore, our study also showed that actual cycling routes included more m/km road where no cycle lane was present which is typical for residential streets in Flanders. In addition, the present study found that actual cycling routes included a larger part of the route where only few buildings with windows on the street were present, which is also an attribute of local roads. As already mentioned above, the presence of buildings with windows on the street refers to social control from people living in the area [22]. Another study among 5-to-18-year-old youth found similar results, participants in that study agreeing that ‘walkers and bikers on the streets in my neighbourhood can easily be seen by people in their homes’ were less likely to use active transport to school [39]. These findings could be explained by the fact that adolescents perceive cycling along local roads with lower speed limits as more important than potential social control from residents. Among adults, Winters et al. [16] also found that cyclists spent most of their travel distance along local roads. In the present study, arterial roads, such as road types which consist of two separate roads each divided in two lanes each direction, were avoided. A number of previous studies also found that cyclists avoid busy, arterial roads [15, 16, 40] and roads with high traffic speed [41]. In Flanders, if any type of cycle path is available along these busy roads it is typically a small cycle path separated from traffic by white lines, which explains why the present study found that this type of cycle path is less present along actual cycling routes.

With regard to walking/cycling roads that are not accessible for motorised traffic, no significant difference in presence along actual and shortest cycling routes was found. Nevertheless, a previous study among adults found that cyclists spent more time on off-street paths [16]. In addition, among 10-to-15-year-old US girls, it was found that the presence of walking/cycling trails in the neighbourhood was associated with higher levels of active transport to school [39]. Although, in the present study, these walking/cycling roads occurred relatively frequently along actual cycling routes (on average for 89 m/km), shortest cycling routes also included some amount of walking/cycling roads (62 m/km) since the city of Ghent already provides an extensive network of walking/cycling roads. These walking/cycling roads often serve as shortcuts for pedestrians and cyclists. This could explain why no significant difference between actual and shortest cycling routes was found for walking/cycling roads in this study.

### Practical implications

Based on the findings of the present study, some recommendations for policy and practice can be formulated. The present study showed that adolescents mainly choose the (perceived) shortest route to cycle for transport and that adolescents frequently use walking/cycling roads that are not accessible for motorised traffic. It might thus be important for local authorities to provide walking/cycling roads that are not accessible for motorised traffic and could serve as shortcuts for cyclists [42]. These shortcuts free from motorised traffic also meet the preference of adolescents to avoid cycling along arterial roads. However, since it is not always possible for an individual to avoid to cycle along busy, arterial roads, these roads should be made more bike-friendly by providing adequate cycling infrastructure.

### Strengths and limitations

A first strength of the present study was that the evaluated routes were actual cycling routes which were objectively recorded using a GPS device. Using objective GPS data limits recall bias related to route choice. Particularly for young people such as adolescents it may be difficult to recall and indicate on a map which route they took at a particular moment. Second, information obtained by the structured one-on-one interviews enabled to correct trip mode when this was misclassified by PALMS (e.g. when a car trip was classified as a bicycle trip due to traffic congestion). Furthermore, this was the first study to collect subjective information regarding route choice via one-on-one interviews, and combine this with audits. This allowed participants to indicate their actual reason for choosing a particular route. Third, the presence of physical environmental characteristics along the routes was measured objectively using a tool (EGA-Cycling) that showed acceptable reliability and validity [22], which limits the bias of results compared to self-reported questionnaires. Nevertheless, some limitations should be acknowledged. First, virtual audit tools showed to be less accurate for measuring micro-environmental features. However, Google Street View showed to be more accurate in measuring micro-environmental features compared to other virtual audit tools [28]. Nevertheless, a discrepancy in physical environmental factors may exist between the Google Street View images and the period in which GPS data were collected. The Google Street View images ranged from March 2009 till April 2015, whereas participants’ GPS data were collected between September and December 2015. Second, the sample size was relatively small as only 38 actual cycling routes (spread over 22 adolescents) that were not the shortest cycling route could be identified and were evaluated. A small sample size increases the likelihood of a type II error and, thus, the chance that an effect was not detected when there was one to be detected. Third, some characteristics (i.e. poor visibility when crossing a street, parking facilities on adjacent parking) were only present along a few street segments. This resulted in wide 95% confidence intervals due to insufficient variability. Fourth, as data collection took place among adolescents attending secondary schools in the city of Ghent, the majority of cycling trips were performed in a (sub)urban area. Thus, results cannot be generalized to rural areas where less alternative routes are available because of a less dense street network. Fifth, because of the limited time window of the study (i.e. 2 days of data per participant), it is difficult to draw generalizable conclusions regarding the impact of the physical environment on adolescents’ route choice while cycling. Sixth, the majority of adolescents was of a higher SES family, which could have influenced the results. Previous research showed that children and adolescents living in lower SES neighbourhoods perceive the neighbourhood environment as less attractive and safe, and more often report heavy traffic in their neighbourhood compared to those living in higher SES neighbourhoods [43, 44]. It is thus possible that adolescents from lower SES families attach importance to other factors on their cycling route compared to adolescents from higher SES families. Thus, caution is needed when generalizing results to the overall adolescent population. Seventh, data were collected during autumn/winter which may have influenced the results. Bad weather and less hours of daylight may influence adolescents’ choice of transport mode and route choice. Finally, results of the present study do not enable to draw conclusions regarding non-cyclists.

### Recommendations for future research

Since data collection was very time-consuming and the burden on participants was rather high, future studies should consider to make use of dedicated smartphone applications to identify adolescents’ actual cycling routes. Adolescents generally carry their smartphones with them during the day, thus running dedicated mobile apps may be less considered as a burden compared to wearing portable GPS-devices. The use of dedicated smartphone applications would enable to include a larger sample and would allow to track adolescents’ mobility patterns over a longer time period [45]. Thus, this method has the advantage that much more actual cycling routes can be identified in more diverse areas. The introduction of smartphones with a longer battery life and a higher storage space and memory capacity should be able to facilitate this type of data collection. Nevertheless, this would imply a huge burden on the researchers to audit such a large set of cycling routes. Geographic Information Systems (GIS) could be used instead, since GIS makes use of existing data sources (e.g. governmental data sources) to measure physical environmental characteristics that have some spatial reference [46]. However, since for some locations (i.e. rural or suburban areas) GIS data may not be available [46] and micro-environmental factors are also not commonly available in GIS databases [46, 47], virtual or on-street audits may be used to complement GIS data where needed. In addition, future studies should consider to investigate potential moderators (e.g. individual and social environmental factors) of the relationships between the presence of physical environmental factors and adolescents’ route choice while cycling for transport. It may be interesting to investigate whether the relationship between the presence of certain physical environmental factors and adolescents’ preference for a certain cycling route is moderated by, for example, psychosocial factors. It could be that adolescents with lower self-efficacy or less social models for active transport attach importance to other physical environmental factors when choosing a cycle route compared to adolescents with higher self-efficacy or more social models for active transport. Results of the present study only enable to draw conclusions for the general adolescent population, no specific conclusions for subgroups of adolescents (e.g. those with a low psychosocial profile towards cycling for transport or the least regular cyclists) can be drawn. Since this study was one of the first exploring the factors associated with route choice among adolescent cyclists, results are valuable. However, future studies may consider to conduct moderation analyses among larger samples in order to be able to formulate recommendations to target specific subgroups of adolescents. More research investigating adolescents’ route choice for cycling is needed. In order to be able to formulate recommendations regarding which factors may stimulate adolescents to cycle for transport, future studies should also investigate which factors along a route are important among non-cyclists. An experimental study which aimed to investigate adolescents’ preferences towards cycling for transport using manipulated photographs, showed that the least regular cyclists in that study attached most importance to cycling distance when indicating which route they preferred to cycle along [48]. The most regular cyclists in that study attached most importance to being able to cycle together with a friend. However, no associations with actual participation in cycling for transport were investigated in that study.

## Conclusions

For 73.1% of the cycling trips, participants took the shortest route possible which confirmed the importance of cycling distance for adolescents. When not taking the shortest cycling route, adolescents avoided to cycle for transport along arterial roads with a small cycle lane separated from traffic by white lines. Local roads with a speed limit of 30 km/h in an area with a low land use mix where few commercial destinations are located were more frequently used, even when no cycle lane was available. In general, the ability to cycle along quiet, local roads overruled the importance of all other physical environmental factors besides distance. Local authorities should provide shortcuts free from motorised traffic in order to meet the preference of adolescents to cycle along the shortest route and to avoid cycling along busy, arterial roads. In addition, it may also be important to provide adequate cycling infrastructure along busy, arterial roads since these roads cannot always be avoided.

## Additional files

**Additional file 1.** Structured one-on-one interview.

**Additional file 2.** EGA-Cycling checklist.
