# Supplementary material for: Differences in physical environmental characteristics between adolescents’ actual and shortest cycling routes: a study using a Google Street View-based audit
Source: Int J Health Geogr. 2018 May 29;17:16. doi: 10.1186/s12942-018-0136-x (PMC5975511; doi:10.1186/s12942-018-0136-x)
Supplement: Supplementary file 1 — Additional file 1. Structured one-on-one interview. [file 12942_2018_136_MOESM1_ESM.docx]

**Additional file 1 – Structured one-on-one interview**

| *Transport mode* | *Reason route choice* |
| --- | --- |
| 1. walking | 1. shortest/fastest route |
| 2. cycling/skateboard/… | 2. possibility to walk/cycle with friend(s)/classmate(s)/… |
| 3. moped | 3. presence of a walking or cycling path |
| 4. car/motorcycle | 4. walking or cycling path well-separated from traffic |
| 5. public transport | 5. well-maintained walking or cycling path |
|  | 6. wide walking or cycling path |
|  | 7. well-lighted walking or cycling path |
|  | 8. few traffic lights which force me to stop regularly |
|  | 9. safe crossings |
|  | 10. calm traffic |
|  | 11. nice environment (e.g. lot of greenery) |
|  | 12. presence of a sports ground/playground |
|  | 13. shop/… which I had to visit on the route |
|  | 14. few hills or bridges |
|  | 15. parents want me to take this route |
|  | 16. clearly indicated where people should walk/cycle |
|  | 17. presence of other people makes me feel safe |
|  | 18. clean/few car exhausts |
|  | 19. other |
