# Supplementary material for: Differences in physical environmental characteristics between adolescents’ actual and shortest cycling routes: a study using a Google Street View-based audit
Source: Int J Health Geogr. 2018 May 29;17:16. doi: 10.1186/s12942-018-0136-x (PMC5975511; doi:10.1186/s12942-018-0136-x)
Supplement: Supplementary file 2 — Additional file 2. EGA-Cycling checklist. [file 12942_2018_136_MOESM2_ESM.docx]

**Additional file 2 - EGA-Cycling checklist**

**Date** ………………………………  **Auditor ID** …………..……….

**Participant ID** …………………………………………………………..

**Route #** …………………………. **actual/shortest**

**urban/semi-urban/rural**

**Segment #** …………………….. **Distance** …….…………… m

***Start*** ………………………………………………………………………

***End***…………………………………………………………………………

**Date stamp GSV** ………………………………………………………

*Does this segment consist of a walking/cycling road (a separate road only accessible for non-motorised traffic)?*

- *No (🡪 proceed on this page)*
- *Yes (🡪 proceed with the walking/cycling road related checklist)*

**LAND USE**

1. Are residential and non-residential land uses visible in this segment?
   - No
   - Yes
   - Not applicable
2. What types of buildings are visible in this segment?
   - Single buildings
   - Closed or semi-detached buildings
   - Apartment buildings
   - Not applicable
3. Are commercial destinations visible in this segment (restaurant, shop, tank station,….)?
   - No
   - Yes
4. Is heavy industry visible in this segment (industrial sites)?
   - No
   - Yes
5. Are public destinations visible in this segment (school, police station, bus stop,…)?
   - No
   - Yes
6. Are recreational destinations visible in this segment (fitness, playground,…)?
   - No
   - Yes
7. Are natural features visible in this segment (river, lake,…)?
   - No
   - Yes
8. Is this segment characterized by an open or closed view?
   - Open view
   - Not open/closed view
   - Closed view

**characteristics street segment**

**GENERAL CHARACTERISTICS**

1. What is the road type?
   - One road for one-direction-traffic
   - One road not divided into lanes
   - One road divided in one lane each direction
   - One road divided in two lanes each direction
   - Two roads divided in one lane each direction
   - Two roads divided in two lanes each direction
2. What is the posted speed limit on this segment?
   - 30 km/h
   - 50 km/h
   - 70 km/h
   - 90 km/h
3. Are there measures on this segment that can slow down traffic?
   - No
   - Yes

Mark all that apply:

- - Roundabout
  - Traffic light
  - Speed bump
  - Speed ramp
  - Traffic slalom
  - Lane narrowing

1. How many side streets are present along this street segment?

……………………………………………. side streets

1. How many intersections are present along this street segment?

……………………….…………………… intersections

1. Are there measures on this segment that make it easier for pedestrians/cyclists to cross over?
   - No
   - Yes

Mark all that apply:

- - Crosswalk
  - Marked crosswalk for cyclists
  - Traffic lights
  - Traffic island
  - Curb extension
  - Underpass/bridge for pedestrians or cyclists
  - Bike box

1. Is there poor visibility at the corners, around roundabouts, or from parked cars when crossing (a side street/intersection)?

- No
- Yes
- Not applicable

1. Is the street segment well maintained?
   - No
   - Yes
2. Are streetlights present in this street segment?
   - No
   - Yes
3. What type of vehicle parking facilities is provided in this street segment?
   - On street
   - Next to the street (front yard, adjacent piece of land)
   - On adjacent parking
   - On separate parking
   - No parking
4. How steep or hilly is this segment?
   - Flat
   - Gentle slope
   - Moderate slope
   - Steep slope
5. Are there swerving alternatives for cyclists (front yard,…)?
   - No
   - Yes
6. How many buildings have windows on the street side to have sight on cyclists?
   - No buildings with windows on street side
   - Few buildings with windows on street side
   - Many buildings with windows on street side
7. How many buildings have driveways where vehicles suddenly can pop up?
   - No driveways
   - Approx. 25% of buildings have one driveway
   - Approx. 50% of buildings have one driveway
   - Most buildings have one driveway
8. How many buildings have garage doors facing the street?
   - No garages
   - Approx. 25% of buildings have one garage
   - Approx. 50% of buildings have one garage
   - Most buildings have one garage

**CYCLING FACILITIES**

1. What type of cycle lane is visible in this segment?
   - Cycle lane separated from the road
   - Adjoining cycle lane (slightly increased)
   - Cycle lane is part of the road (white lines)
   - Non-compulsory cycle lane or of a different colour
   - No cycle lane 🡺 *proceed to (6)*
2. What is the width of the cycle lane?
   - Small (space for 1 cyclist)
   - Wide (space for 2 cyclists)
3. Is it a two-way cycle lane?
   - No
   - Yes
4. Is the cycle lane well maintained?
   - No
   - Yes
5. Does lighting cover the cycle lane area?
   - No
   - Yes
6. What is the surface of the cycle lane? (If no cycle lane is present, evaluate the road)
   - Bitumen
   - Continuous concrete
   - Paving bricks
   - Concrete slabs
   - Cobblestones
   - Gravel
7. What is the path condition and smoothness?
   - Poor (a lot of bumps, cracks, holes)
   - Moderate (some bumps, cracks, holes)
   - Good (very few bumps, cracks, holes)

**PEDESTRIAN FACILITIES**

1. Is there a sidewalk visible in this segment?
   - No 🡺 *proceed to ‘aesthetics’*
   - Yes
2. Is the sidewalk well maintained?
   - No
   - Yes
3. Does lighting cover the sidewalk area?
   - No
   - Yes

**aesthetics**

1. Are trees visible in this segment (e.g. avenue of trees)?
   - No
   - Yes
2. Are attractive buildings visible in this segment (historical buildings, architectural design, building variety)?
   - No
   - Yes
3. Are the buildings well maintained in this segment?

- No
- Yes
- Not applicable

1. Are front yards visible in this segment?

- No
- Yes

1. Are the front yards well maintained?

- No
- Yes
- Not applicable

1. Are attractive natural features visible in this segment?

- No
- Yes

1. Are graffiti and litter apparent in this segment?

- No
- Yes
